# *ECPPF* (*E**2F1*, *C**CNA2*, *P**OLE*, *P**PP2R1A*, *F**BXW7*) stratification: Profiling high-risk subtypes of histomorphologically low-risk and treatment-insensitive endometrioid endometrial cancer

**DOI:** 10.1371/journal.pone.0278408

**Published:** 2022-12-01

**Authors:** Jesus Gonzalez-Bosquet, S. John Weroha, Jamie N. Bakkum-Gamez, Amy L. Weaver, Michaela E. McGree, Sean C. Dowdy, Abimbola O. Famuyide, Benjamin R. Kipp, Kevin C. Halling, Siddhartha Yadav, Fergus J. Couch, Karl C. Podratz

**Affiliations:** 1 Department of Obstetrics and Gynecology, University of Iowa, Iowa City, Iowa, United States of America; 2 Division of Medical Oncology, Mayo Clinic, Rochester, Minnesota, United States of America; 3 Division of Clinical Pharmacology, Mayo Clinic, Rochester, Minnesota, United States of America; 4 Mayo Clinic Cancer Center, Mayo Clinic, Rochester, Minnesota, United States of America; 5 Department of Obstetrics and Gynecology, Mayo Clinic, Rochester, Minnesota, United States of America; 6 Department of Quantitative Health Sciences, Mayo Clinic, Rochester, Minnesota, United States of America; 7 Department of Laboratory Medicine and Pathology, Mayo Clinic, Rochester, Minnesota, United States of America; 8 Department of Clinical Genomics, Mayo Clinic, Rochester, Minnesota, United States of America; 9 Department of Biochemistry and Molecular Biology, Mayo Clinic, Rochester, Minnesota, United States of America; Avera Research Institute, UNITED STATES

## Abstract

In endometrial cancer, occult high-risk subtypes (rooted in histomorphologically low-risk disease) with insensitivity to adjuvant therapies impede improvements in therapeutic efficacy. Therefore, we aimed to assess the ability of molecular high-risk (MHR) and low-risk (MLR) *ECPPF* (*E**2F1*, *C**CNA2*, *P**OLE*, *P**PP2R1A*, *F**BXW7*) stratification to profile recurrence in early, low-risk endometrioid endometrial cancer (EEC) and insensitivity to platinum-based chemotherapy or radiotherapy (or both) in high-risk EEC. Using The Cancer Genome Atlas endometrial cancer database, we identified 192 EEC cases with available DNA sequencing and RNA expression data. Molecular parameters were integrated with clinicopathologic risk factors and adverse surveillance events. MHR was defined as high (-H) *CCNA2* or *E2F1* log_2_ expression (≥2.75), *PPP2R1A* mutations (-mu), or *FBXW7*mu; MLR was defined as low (-L) *CCNA2* and *E2F1* log_2_ expression (<2.75). We assessed 164 cases, plus another 28 with *POLE*mu for favorable-outcomes comparisons. MHR and MLR had significantly different progression-free survival (PFS) rates (*P* < .001), independent of traditional risk factors (eg, *TP53*mu), except for stage IV disease. PFS of *CCNA2*-L/*E2F1*-L paralleled that of *POLE*mu. *ECPPF* status stratified responses to adjuvant therapy in stage III-IV EEC (*P* < .01) and profiled stage I, grade 1–2 cases with risk of recurrence (*P* < .001). MHR was associated with *CTNNB1*mu-linked treatment failures (*P* < .001). Expression of homologous recombination repair (HR) and cell cycle genes was significantly elevated in *CCNA2*-H/*E2F1*-H compared with *CCNA2*-L/*E2F1*-L (*P*<1.0E-10), suggesting that HR deficiencies may underlie the favorable PFS in MLR. HRmu were detected in 20.7%. No treatment failures were observed in high-grade or advanced EEC with HRmu (*P* = .02). Favorable PFS in clinically high-risk EEC was associated with HRmu and MLR *ECPPF* (*P* < .001). In summary, MLR *ECPPF* and HRmu were associated with therapeutic efficacy in EEC. MHR *ECPPF* was associated with low-risk, early-stage recurrences and insensitivity to adjuvant therapies.

## Introduction

The advent of surgical staging and adjuvant platinum-based chemotherapy (PbCT) in the management of endometrial cancer (EC) occurred 30 years ago, and a plethora of publications addressing their virtues (or lack thereof) have appeared since then. The American Cancer Society reports provide sobering comparisons of estimated cancer statistics for 1991 and 2021—with 33,300 and 66,570 new cases and 5,500 and 12,940 deaths, respectively [1, 2]. From 2008 through 2015, the age-adjusted EC mortality rate was reported to have a 1.9% per annum increase, further stressing the need to characterize therapeutic deficiencies and formulate resolutions [3]. Enhanced knowledge is needed to identify and target vulnerabilities within the oncogenic signaling pathways of EC.

Considering the morphologic similarities to ovarian cancer and high prevalence of *TP53* variants in serous EC (SEC), the adaptation of PbCT from high-grade serous ovarian cancer appears reasonable. However, the efficacy of PbCT is generally predicated on deficiencies in homologous recombination repair (HR) [4], and genomic and epigenomic assessments have shown disparities in HR deficiencies of endometrioid EC (EEC) and SEC compared with high-grade serous ovarian cancer [5, 6]. The frequency of HR variants and HR deficiency is less than 20% in SEC and EEC [7, 8], and unsurprisingly, the responses to PbCT are suboptimal for SEC and high-grade EEC [9–12]. In contrast, low-grade EEC generally appears sensitive to PbCT [12–14].

Impaired apoptosis, cell cycle dysregulation, and enhanced DNA damage repair (DDR) are dominant characteristics associated with radioresistance and chemoresistance in solid tumors [15–17]. Our previous studies integrating EC molecular aberrations identified common characteristics among high-risk EC—specifically, CCNA2-E2F1-CIP2A axis dysregulation, coupled with *PPP2R1A* and *FBXW7* variants, were associated with insensitivity to adjuvant therapy and a poor prognosis [18]. Overexpressed CCNA2 binds to E2F1 and converts it from an apoptotic regulator to a potent transcription activator [19]. *FOXM1*, *CIP2A*, and multiple cell cycle and HR genes harbor E2F1 activation sites [20–22]; FOXM1 reportedly induces several HR genes, and CIP2A is the nexus to the PI3K-AKT pathway [23]. Upregulation of CIP2A or the presence of variants in *PPP2R1A* or *FBXW7* impede the proteasomal degradation of FOXM1 and multiple cell cycle proteins [24, 25]. The activation of *FOXM1* and cell cycle genes, combined with the suppressed degradation of their corresponding proteins, portends overexpression of HR pathway components and insensitivity to DNA-damaging agents in HR-proficient EC [15–17, 20–25].

EEC provides an intriguing model for examining molecular characteristics, including genomic and transcriptomic anomalies, across a spectrum of grades, stages, depths of myometrial invasion (MI), and clinical outcomes. In this study, we assessed the ability of the recently reported *E**2F1*, *C**CNA2*, *P**OLE* mutations (-mu), *P**PP2R1A*mu, and *F**BXW7*mu molecular stratification system [18] (hereafter termed *ECPPF*) to characterize patients with suboptimal outcomes after EEC treatment. We assessed *ECPPF* in the setting of numerous clinicopathologic parameters, HR and prevalent EC-associated variants, and HR, cell cycle, and related gene expression patterns.

## Methods

### Study population

The original report describing the prognostic stratification of EC by *ECPPF* [18] included 239 cases with annotated clinicopathologic, DNA sequencing, RNA expression, and surveillance data that were abstracted from the initial public release of The Cancer Genome Atlas (TCGA) EC database [5]. The prior report was substantially leveraged by the sizeable SEC cohort [18]. The current study (N = 192) was based on the same data set but excluded serous histology to focus on assessment of *ECPPF* stratification in EEC as a function of grade, depth of MI, stage, expression of select genes, and specific, relevant variants. TCGA required surgical staging, but adjuvant therapy was unknown for approximately half the cases. Nevertheless, PbCT and radiotherapy were the predominant adjuvant therapies during the period of patient accrual; 98% of patients receiving chemotherapy received PbCT [18].

TCGA classification defined 4 subgroups: 1) ultramutated (*POLE*mu, exonuclease domain mu); 2) hypermutated (microsatellite unstable); 3) copy number low (CNL) or no specific molecular profile (NSMP); and 4) copy number high (CNH; *TP53*mu/serous-like) [18]. Microsatellite instability (MSI), CNL (NSMP), and CNH were integrated in the EEC *ECPPF* stratification; CNH was defined as *TP53*mu, and CNL (NSMP) was estimated as 192 –(*POLE*mu + MSI + *TP53*mu). *POLE*mu were excluded from analyses but served as a metric of favorable clinical outcome.

### The cancer genome atlas

We downloaded data from the TCGA EEC database [5]. Data were normalized, formatted, and organized for integration and analysis, as described previously [18, 26].

All data collection and processing, including the consenting process, were performed after approval was obtained from each contributing institution’s local institutional review board or ethics committee and in accordance with the TCGA Human Subjects Protection and Data Access Policies, adopted by the National Cancer Institute and the National Human Genome Research Institute.

### Variant analysis

Only validated variants (or TCGA level 3 variants) were used for analysis. Variant information was extracted from exome sequencing data obtained with the Illumina Genome Analyzer GAIIx or HiSeq 2000 sequencing platforms (Illumina, Inc). Silent variants were excluded from the analysis, and only frame-shift insertions and deletions, in-frame insertions or deletions, and missense, nonsense, nonstop, and splice-site variants were included in the study. Two hundred thirty-nine patients from the TCGA EC cohort were reported to have somatic variants in 18,388 unique genes. For our analysis, the number of variants for each selected gene and for each patient were recorded.

### Gene expression

Normalized and log-transformed gene expression data from the TCGA EC database were downloaded as level 3 RNA-sequenced data. As previously described, data were collected by Illumina RNA sequencer HiSeq 2000 platforms and annotated with the hg19 version of the human genome.

Gene expression analyses were performed with R statistical packages for computing and graphics (The R Foundation) [27] and the Bioconductor open-source bioinformatics software package [28].

### Statistical analysis

Data are descriptively summarized with frequencies and percentages for categorical variables and mean (SD) or median (IQR) for continuous variables. Comparisons of gene expression levels between cohorts was evaluated with the 2-sample *t* test. Correlations between expression levels were assessed with the Pearson correlation coefficient. Follow-up was calculated from the date of surgery to the date of first documented progression or last follow-up. Progression-free survival (PFS) was estimated with the Kaplan-Meier method. Cox proportional hazards models were fit to evaluate the association between assessed molecular parameters and the risk of progression; associations were summarized with the hazard ratio, and the corresponding 95% CIs were estimated from the models. All calculated *P* values less than .05 were considered statistically significant. Data were analyzed with SAS software (version 9.4; SAS Institute Inc).

## Results

### Study population demographic characteristics

DNA sequencing and RNA expression data were annotated for 192 EEC cases in the TCGA EC database, which facilitated assessment of molecular aberrations, clinicopathologic risk factors, and adverse surveillance events [5]. *POLE*mu were detected in 28 cases (14.6%). These mutations served as a marker of favorable prognoses [5], and cases with *POLE*mu were excluded from the integrative assessments. The remaining 164 cases constituted our main study cohort (median age, 61 years). In this cohort (Table 1), 124 patients had stage I disease (21 were grade [G] 3); 10 had stage II disease (3 were G3); 23 had stage III disease (6 were G3); and 7 had stage IV disease (6 were G3). *PPP2R1A*mu were present in 15 patients (9.1%), *FBXW7*mu were present in 9 (5.5%), and *TP53*mu were present in 21 (12.8%). When categorizing *TP53*mu prevalence by tumor grade, 1 of 63 G1 cases had *TP53*mu, as did 8 of 65 G2 cases and 12 of 36 G3 cases. When assessing variants in HR genes, including *ATM*, *ATR*, *BRCA1*, *BRCA2*, *PALB2*, *CDK12*, *BARD1*, *NBN*, *CHK1*, and *Rad51*, 1 or more variants were identified in 34 patients (20.7%). Fifty-eight patients (35.4%) had high microsatellite instability (MSI-H) and 85 (51.8%) had CNL (microsatellite stable; NSMP). Recurrences (time from surgery date) were documented for 31 patients (18.9%); for the other 133 patients, the median duration of follow-up was 28.7 months (IQR, 15.9–46.8 months).

**Table 1:** Clinicopathologic parameters, TCGA subgroups, and select variants, stratified by molecular high-risk and low-risk endometrioid endometrial cancer.

Characteristic^a^	*CCNA2*-H/*E2F1*-H (n = 44)	*CCNA2*-L/*E2F1*-L (n = 97)	*FBXW7*mu/*PPP2R1A*mu (n = 23)	Total (n = 164)
Parameter stage				
I	27 (61.4)	78 (80.4)	19 (82.6)	124 (75.6)
II	5 (11.4)	5 (5.2)	19 (82.6)	10 (6.1)
III	7 (15.9)	12 (12.4)	4 (17.4)	23 (14.0)
IV	5 (11.4)	2 (2.1)	0 (0)	7 (4.3)
Histologic grade				
1	7 (15.9)	44 (45.4)	12 (52.2)	63 (38.4)
2	15 (34.1)	42 (43.3)	8 (34.8)	65 (39.6)
3	22 (50.0)	11 (11.3)	3 (13.0)	36 (22.0)
Myometrium invasion				
≤50%	33 (75.0)	75 (77.3)	19 (82.6)	127 (77.4)
>50%	11 (25.0)	22 (22.7)	4 (17.4)	37 (22.6)
TCGA classification subgroup				
*POLE*mu^b^	n = 19	n = 9	n = 17	n = 28
MSI-H	23 (52.3)	26 (26.8)	9 (39.1)	58 (35.4)
CNL (NSMP)	9 (20.5)	62 (63.9)	14 (60.9)	85 (51.8)
*TP53*mu	12 (27.3)	9 (9.3)	0 (0)	21 (12.8)
PIK3CA-AKT-FBXW7 pathway variant				
*PIK3CA*	22 (50.0)	58 (59.8)	13 (56.5)	93 (56.7)
*PTEN*	32 (72.7)	70 (72.2)	12 (52.2)	114 (69.5)
*CTNNB1*	18 (40.9)	29 (29.9)	7 (30.4)	54 (32.9)
*PPP2R1A*	3 (6.8)	12 (12.4)	15 (65.2)^c^	15 (9.1)^c^
*FBXW7*	1 (2.3)	8 (8.2)	9 (39.1)^c^	9 (5.5)^c^
DNA damage repair variant				
*ATR*	4 (9.1)	6 (6.2)	1 (4.3)	11 (6.7)^d^
*ATM*	3 (6.8)	6 (6.2)	1 (4.3)	10 (6.1)^d^
*BRCA2*	4 (9.1)	4 (4.1)	1 (4.3)	9 (5.5)^d^
*BRCA1*	1 (2.3)	3 (3.1)	0 (0)	4 (2.4)^d^
*PALB2*	1 (2.3)	2 (2.1)	0 (0)	3 (1.8)
*CDK12*	1 (2.3)	2 (2.1)	0 (0)	3 (1.8)
*BARD1*	1 (2.3)	1 (1.0)	0 (0)	2 (1.2)
*NBN*	1 (2.3)	1 (1.0)	0 (0)	2 (1.2)
*CHEK1*	1 (2.3)	0 (0)	0 (0)	1 (0.6)
*Rad51*	0 (0)	1 (1.0)	0 (0)	1 (0.6)

Abbreviations: CNL (NSMP), copy number low (no specific molecular profile); -H, high; -L, low; MSI-H, high microsatellite instability; -mu, mutation; TCGA, The Cancer Genome Atlas.

^a^ Data are reported as number of cases (%).

^b^ Prevalence of *POLE*mu cases (excluded from analyses) among high- and low-risk cohorts.

^c^ A single case harbored *PPP2R1A*mu and *FBXW7*mu.

^d^ Among *POLE*mu cases, the prevalence of *ATR*, *ATM*, *BRCA2*, and *BRCA1* variants was 50.0%, 67.9%, 53.6%, and 35.7%, respectively.

### *ECPPF* stratification of EEC

**Fig 1:**
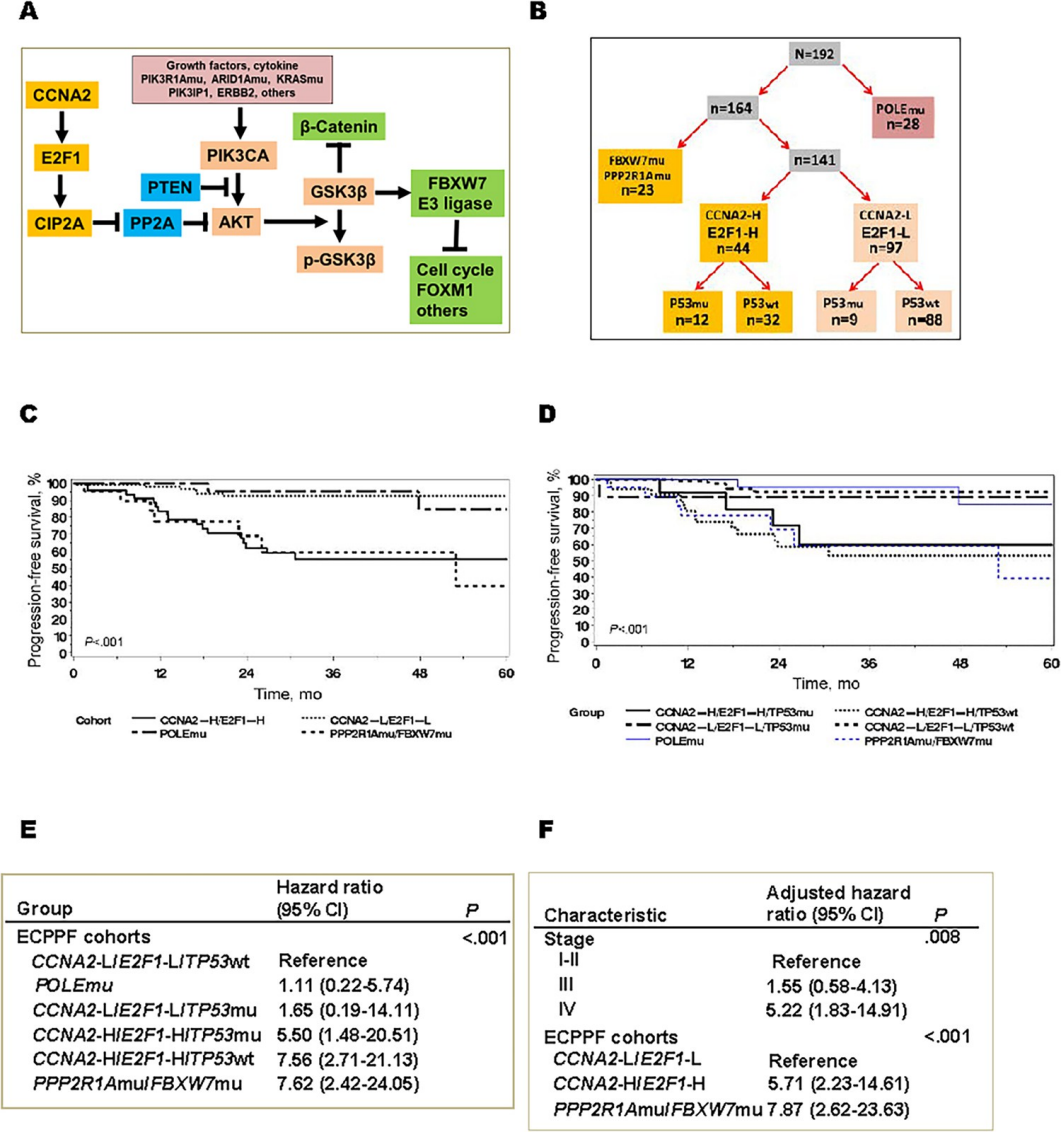
Molecular profile and clinical outcomes. A, Integrated schematic showing the CCNA2-E2F1-CIP2A axis and PI3K-AKT-GSK3β-FBW7 pathway. B, *ECPPF* stratification was used to identify distinct molecular subgroups from a cohort of 192 patients with endometrioid endometrial cancer. The stratification scheme considers *POLE* mutations (-mu), *PPP2R1A*mu, *FBXW7*mu, *CCNA2*-H/*E2F1*-H, and *CCNA2*-L/*E2F1*-L; the latter 2 are stratified by *TP53* status (*TP53*mu and *TP53* wild type [-wt]). Suffixes -H and -L denote high or low gene expression levels, respectively. C, Progression-free survival rates for 4 primary *ECPPF* molecular cohorts. D, Progression-free survival for *ECPPF* molecular cohorts with *CCNA2*-H/*E2F1*-H and *CCNA2*-L/*E2F1*-L, stratified by *TP53*mu and *TP53*wt. E, Cox proportional hazards model with hazard ratios for 6 *ECPPF* molecular cohorts, using *CCNA2*-L/*E2F1*-L/*TP53*wt as the reference. F, A stepwise multivariate analysis considering competing variables with *P* < .20 in the univariate analysis (age, grade, depth of myometrial invasion, stage, high microsatellite instability, no specific molecular profile, *TP53*mu, homologous recombination repair mutations, *CTNNB1*mu, *KRAS*mu, *ARID1A*mu, *PIK3CA*mu, *PTEN*mu, *PIK3R1*mu, *CIP2A* expression, and *ECPPF* cohorts).

*CCNA2* and *E2F1* expression and *PPP2R1A* and *FBXW7* mutations, elements in the CCNA2-E2F1-CIP2A axis and PI3K-AKT pathways, respectively, form the basis of the *ECPPF* stratification scheme (Fig 1A and 1B). PFS was assessed within strata defined by previously reported [18] *ECPPF-*discriminating molecular parameters: high (-H) *CCNA2* or *E2F1* log_2_ expression (≥2.75; termed *CCNA2*-H/*E2F1*-H) vs low (-L) *CCNA2* or *E2F1* log_2_ expression (<2.75; termed *CCNA2*-L/*E2F1*-L) and variants in *PPP2R1A* or *FBXW7* (or both). These *ECPPF* cohorts showed significantly different clinical outcomes (*P* < .001) (Fig 1C). PFS for patients with *CCNA2*-L/*E2F1*-L and *POLE*mu tumors appeared nearly equivalent. These results suggest that *ECPPF* status profiles patients with different EEC responses to standard therapy.

Because *TP53*mu has been associated with poorer EC clinical outcomes [5], we evaluated its integration in *ECPPF*. Notably, *TP53*mu (n = 21) were not observed with *PPP2R1*mu and *FBXW7*mu (n = 23) and appeared to be mutually exclusive variants (*P* = .01). *TP53* status (ie, mutant vs wild type [-wt]) did not appear to further stratify either *CCNA2*-L/*E2F1*-L or *CCNA2*-H/*E2F1*-H cohorts (Fig 1D). Cox proportional modeling strengthened these observations, suggesting that *ECPPF-*stratified oncologic outcomes were independent of *TP53*mu (Fig 1E). A stepwise multivariate analysis considering the variables of age, grade, depth of MI, stage, MSI-H, CNL (NSMP), *TP53*mu, HRmu, *CTNNB1*mu, *KRAS*mu, *ARID1A*mu, *PIK3CA*mu, *PTEN*mu, *PIK3R1*mu, *CIP2A* expression, and *ECPPF* parameters (all *P* < .20 in a univariate analysis) showed independent significance only for *ECPPF* variables and stage IV disease (vs stage I-II disease) (Fig 1F). These data suggest that clinical outcomes in patients with EEC can be stratified by *ECPPF*.

### Cell cycle and DDR gene expression in *ECPPF* strata

Disparate responses of *ECPPF* subgroups to adjuvant therapy suggested potentially divergent levels of DDR and cell cycle genes, as annotated within the *CCNA2*-H/*E2F1*-H, *CCNA2*-L/*E2F1*-L, and *PPP2R1A*mu/*FBXW7*mu cohorts (Table 2). We observed striking, significant differences between the *CCNA2*-H*/E2F1*-H and *CCNA2*-L/*E2F1*-L cohorts in expression of numerous cell cycle, DDR, and historically prognostic genes. Notwithstanding the substantial difference in PFS between the *CCNA2*-L/*E2F1*-L and *PPP2R1A*mu/*FBXW7*mu cohorts (Fig 1C), their abridged clinicopathologic profiles (Table 1), and transcriptomic profiles (Table 2, cohort B vs cohort C) were remarkably similar. The extremely low log_2_ mRNA expression of DDR and cell cycle genes in the *CCNA2*-L/*E2F1*-L cases (Table 2) may signal deficiencies in HR function and thus favorable responses to PbCT and radiotherapy [29–31]. In contrast, the high expression of these genes in *CCNA2*-H/*E2F1*-H compared with *CCNA2*-L/*E2F1*-L suggests enhanced HR proficiency, and in the absence of HRmu, insensitivity of *CCNA2*-H/*E2F1*-H to PbCT or radiotherapy (or both) is likely (Fig 1C).

**Table 2:** Gene log_2_ mRNA expression as a function of molecular high- and low-risk endometrioid endometrial cancer cohorts^a^.

Gene	Cohort A: *CCNA2*-H/*E2F1*-H (n = 44)	Cohort B: *CCNA2*-L/*E2F1*-L (n = 97)	Cohort C: *FBXW7*mu/*PPP2R1A*mu (n = 23)	*P* value, comparison of cohorts	
				A to B	B to C
***CCNA2-E2F1-CIP2A* axis**					
*CCNA2*	3.215 (0.787)	1.573 (0.829)	1.910 (0.894)	8.247E-21	.09
*E2F1*	3.217 (1.055)	1.378 (0.712)	1.796 (0.755)	1.349E-23	.013
*CIP2A*	2.400 (0.963)	0.895 (0.934)	0.955 (0.994)	5.303E-15	.78
**Cell cycle genes**					
*CCNB1*	5.297 (0.707)	3.926 (0.686)	4.255 (0.722)	2.473E-20	.043
*CCNB2*	4.706 (0.697)	3.302 (0.789)	3.579 (0.839)	1.993E-18	.14
*CCNE1*	3.251 (1.274)	2.117 (1.220)	1.933 (1.013)	1.392E-06	.50
*AURKA*	3.108 (0.837)	1.644 (0.754)	1.826 (0.699)	6.756E-19	.29
*TPX2*	4.226 (0.741)	2.653 (0.778)	2.893 (0.761)	2.259E-21	.19
*PLK1*	4.518 (0.828)	2.823 (0.795)	3.186 (0.878)	4.021E-22	.06
*ESPL1*	1.971 (0.816)	0.253 (0.844)	0.429 (0.864)	2.011E-21	.38
*CHEK1*	2.634 (0.698)	1.646 (0.494)	1.881 (0.593)	4.513E-17	.05
***FOXM1* and DNA damage repair genes**					
*FOXM1*	4.393 (0.735)	2.802 (0.709)	3.024 (0.665)	9.553E-24	.17
*BRCA1*	1.828 (0.773)	0.714 (0.774)	0.994 (0.738)	6.530E-13	.12
*BRCA2*	−0.749 (1.233)	−2.288 (1.269)	−2.090 (1.141)	4.141E-10	.50
*Rad51*	2.209 (0.748)	0.811 (0.779)	1.072 (0.944)	4.500E-18	.17
*BRIP1*	0.104 (0.893)	−1.148 (0.870)	−0.955 (0.803)	9.950E-13	.33
*EXO1*	1.487 (0.861)	−0.016 (1.039)	0.445 (0.968)	5.303E-14	.06
*MRE11A*	1.120 (0.761)	0.672 (0.730)	0.530 (0.796)	.001	.41
*Rad50*	2.222 (0.780)	2.069 (0.634)	1.840 (0.572)	.22	.12
*NBN*	2.732 (0.830)	2.387 (0.830)	2.321 (0.790)	.024	.73
*ATR*	1.700 (0.574)	1.669 (0.504)	1.461 (0.403)	.75	.07
*ATM*	4.329 (0.708)	4.316 (0.682)	4.212 (0.784)	.92	.53
*SKP2*	2.916 (0.795)	1.922 (0.751)	1.959 (0.710)	4.500E-11	.83
*PARP1*	5.431 (0.621)	4.721 (0.795)	4.886 (0.804)	5.925E-07	.38
**Historically prognostic markers**					
*ATAD2*	2.337 (0.869)	0.833 (0.915)	1.162 (0.754)	5.173E-16	.11
*BIRC5*	5.883 (0.923)	4.307 (0.780)	4.571 (0.733)	2.581E-19	.14
*EZH2*	2.993 (0.622)	2.165 (0.628)	2.103 (0.632)	2.407E-11	.67
*L1CAM*	1.901 (4.754)	0.306 (0.631)	0.396 (1.027)	.0014	.59
*STMN*	7.121 (1.093)	6.288 (0.951)	6.479 (1.317)	9.617E-06	.43

Abbreviations: -H, high; -L, low; -mu, mutation(s).

^a^ Gene expression data (normalized and log-transformed) are shown as mean (SD).

**Fig 2:**
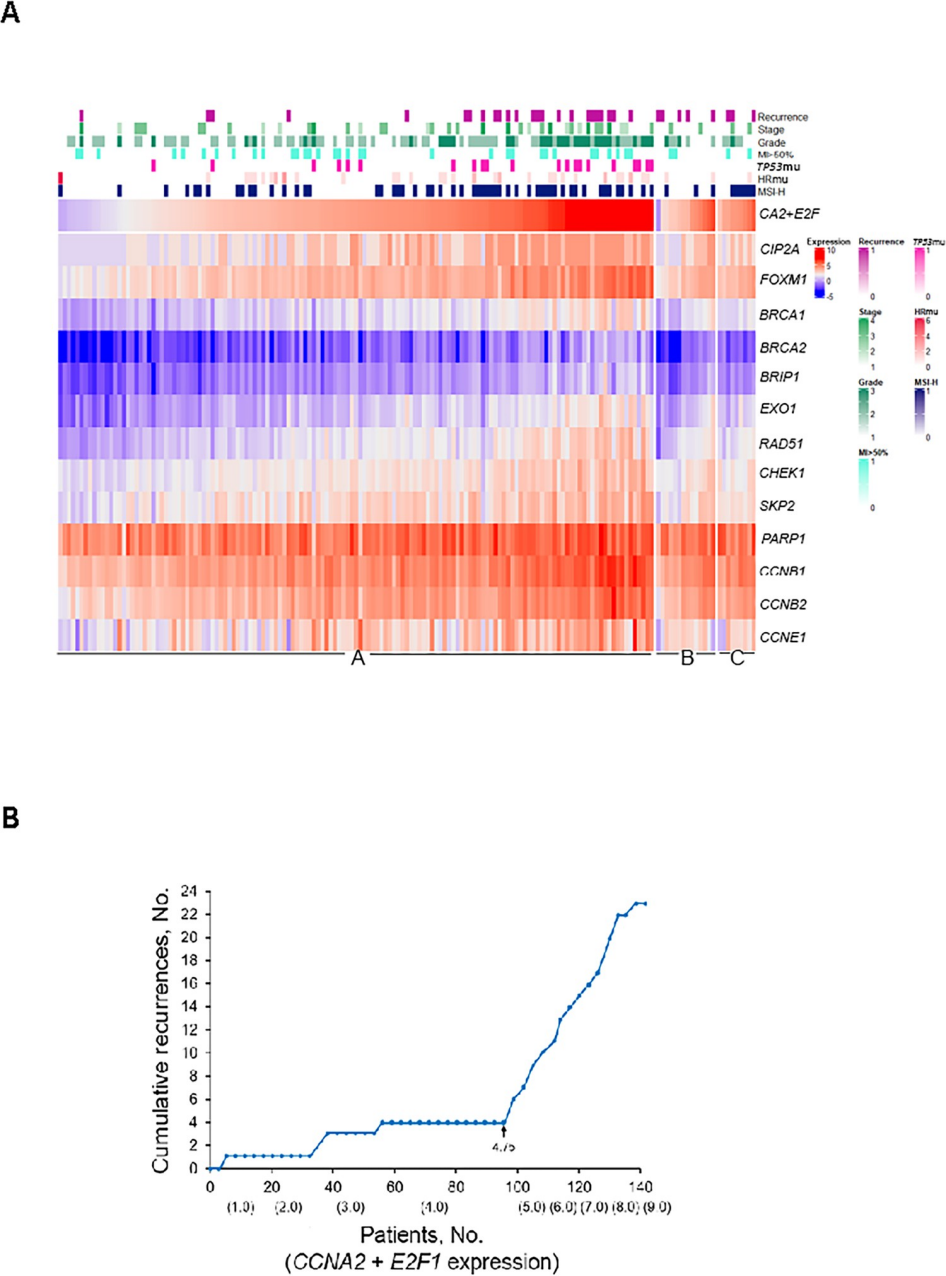
Gene expression and treatment failures. A, The heat map shows distribution of recurrences, stage, myometrial invasion (MI) >50%, mutation (-mu) in *TP53* (*TP53*mu), homologous recombination repair mutations (HRmu), and high microsatellite instability (MSI-H), according to the increasing quantitative sum of *CCNA2* and *E2F1* log_2_ mRNA expression (*CA2*+*E2F*). *CIP2A*, *FOXM1*, *SKP2*, *PARP1*, and HR and cell cycle gene mRNA expression levels also are shown as a function of increasing expression of *CA2*+*E2F*. Group A was defined as the *CA2*+*E2F* cohort minus *PPP2R1A*mu and *FBXW7*mu cohorts (n = 141); group B was the *PPP2R1A*mu cohort (n = 14), and group C was the *FBXW7*mu cohort (n = 9). Patients with *POLE*mu were excluded from this analysis. B, Cumulative recurrences are shown as a function of the expression sum of *CCNA2* and *E2F1* (*CA2*+*E2F*).

The quantitative combined expression (log_2_) of *CCNA2* and *E2F1* (*CA2*+*E2F*) was compared with cell cycle and DDR gene expression levels to further weigh the interdependence of these transcriptomic markers. As *CA2*+*E2F* expression progressively increased, expression of *CIP2A*, *FOXM1*, cell cycle, and HR genes showed a near-parallel increase (Fig 2A). We determined the Pearson correlation coefficients for expression of *CA2*+*E2F* and various genes: with *CIP2A*, *r* = 0.796; with *FOXM1*, *r* = 0.918; with cell cycle genes, *r* ranged from 0.549 to 0.859; and with HR genes, *r* ranged from 0.683 to 0.846. Increasing *CA2*+*E2F* expression was associated with an elevated prevalence of G3 histology, *TP53*mu, and recurrences. If we excluded patients with *PPP2R1A*mu/*FBXW7*mu, cumulative recurrences sharply increased when *CA2*+*E2F* expression exceeded 4.75 (Fig 2B). These results support *ECPPF*’s potential to profile treatment failure for low-risk tumors and treatment insensitivity for high-risk tumors.

### Association of molecular high-risk *ECPPF* with recurrences in low-risk, stage I EEC

**Fig 3:**
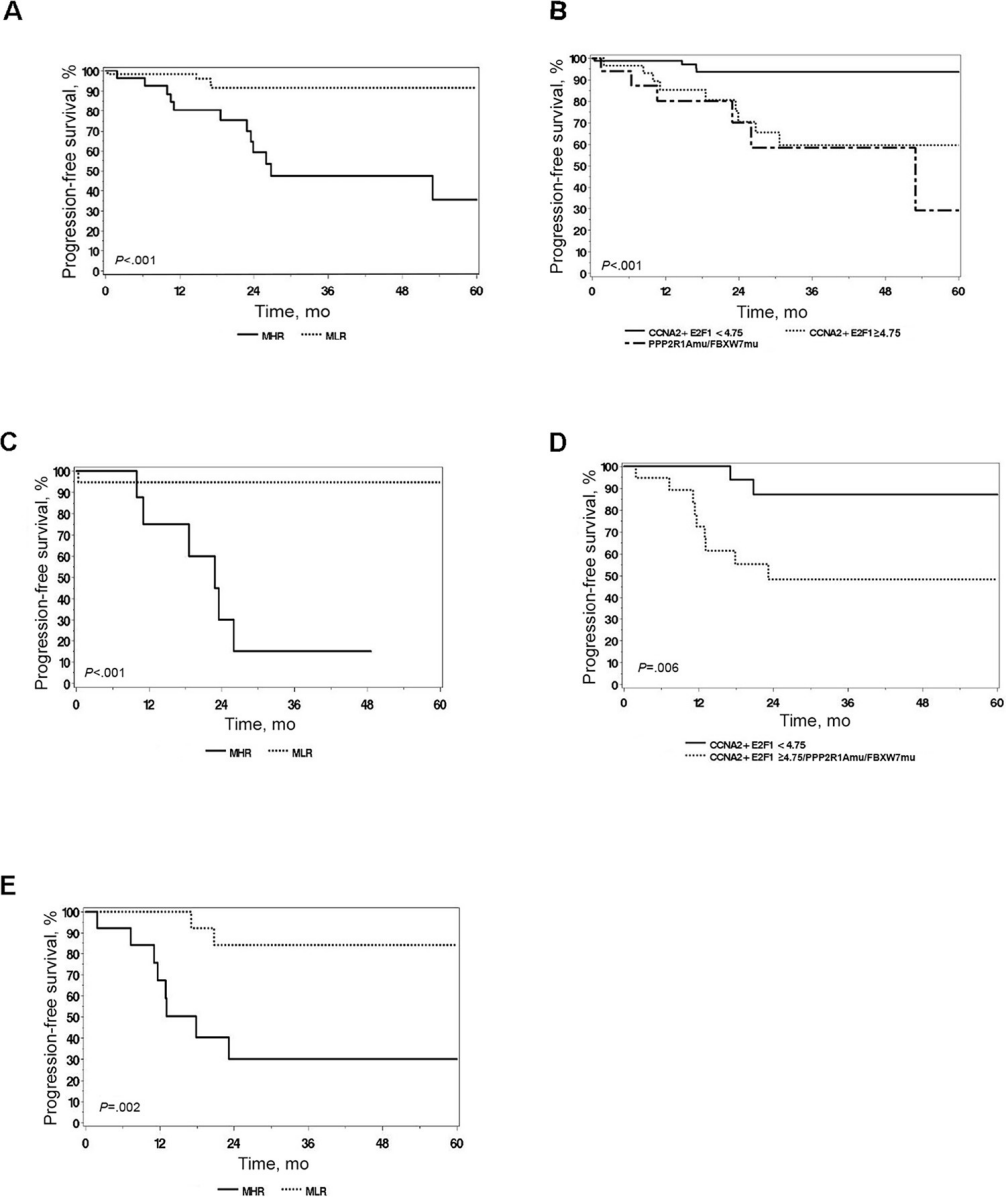
*ECPPF* stratification of outcomes in endometrioid endometrial cancer. A, Progression-free survival for 85 patients with stage I, grade (G) 1 or G2, ≤50% myometrial invasion (MI) disease, stratified by *ECPPF* as having molecular low risk (MLR; defined as *CCNA2* and *E2F1* [*CA2*+*E2F*] log_2_ expression <4.75, *PPP2R1A* wild type [-wt], and *FBXW7*wt [n = 56]) or molecular high risk (MHR; defined as *CA2*+*E2F* log_2_ expression ≥4.75, *PPP2R1A* mutation [-mu], or *FBXW7*mu [n = 29]). B, Progression-free survival for 122 patients with stage I-II, G1-G2, <75% MI disease and patients with G3, <50% MI disease, stratified according to *ECPPF* risk cohorts *CA2*+*E2F* <4.75 (n = 75), *CA2*+*E2F* ≥4.75 (n = 29), and *PPP2R1A*mu/*FBXW7*mu (n = 18). C, Progression-free survival for 28 patients with stage I, G1-G2, ≤50% MI disease and *CTNNB1*mu, stratified according to MLR (n = 19) and MHR (n = 9) *ECPPF*. D, Progression-free survival for 42 patients with stage III-IV disease, stage I-II, G2, >75% MI disease, and G3, >50% MI disease, stratified according to MLR (n = 22) and MHR (n = 14). E, Progression-free survival for 30 patients with stage III-IV, stratified according to MLR (n = 16) and MHR (n = 14) *ECPPF*.

*ECPPF*’s ability to categorize patients with EEC by clinical outcomes, independent of early stage and histologic grade, suggested that *ECPPF* might be able to identify early-stage, low-grade cases with heightened risk of occult extrauterine disease. Clinical outcomes were annotated according to stage (and by grade for stage I disease) and were assessed as a function of molecular low-risk *ECPPF* (MLR; defined as *CA2*+*E2F* <4.74) and molecular high-risk *ECPPF* (MHR; defined as *CA2+E2F* ≥4.75, *PPP2R1A*mu, and/or *FBXW7*mu). Among 103 patients with stage I, G1 or G2 disease, occult extrauterine disease that escaped surgical detection was subsequently documented in 20 patients (19.4%; 5 were MLR, 15 were MHR). When categorizing these patients by grade, 6 of 52 (11.5%) patients with G1 EEC had occult disease, as did 14 of 51 (27.5%) with G2 EEC. Sixty-eight patients (66%) with early-stage, low-grade EEC were categorized as being MLR and 35 (34%) as MHR; the estimated 3-year PFS was significantly different between groups (MLR, 93.0%; MHR, 40.9%; *P* < .001). The corresponding hazard ratio for MHR (using MLR as the reference) was 10.28 (95% CI, 3.42–30.97; *P* < .001). For the 85 patients with stage I, G1 or G2, and ≤50% MI disease, who rarely were candidates for adjuvant therapy, *ECPPF* stratification showed divergent PFS outcomes (*P* < .001) (Fig 3A). The corresponding hazard ratio (95% CI) for MHR (using MLR as the reference) was 7.50 (95% CI, 2.43–23.13; *P* < .001).

*ECPPF* stratification similarly discriminated between outcomes when the cohort was expanded to patients with stage I or II, G1 or G2 disease, and <75% MI and patients with G3 and <50% MI (*P* < .001) (Fig 3B). The corresponding hazard ratio for *CA2*+*E2F* ≥4.75 (using *CA2*+*E2F* <4.75 as the reference) was 6.34 (95% CI, 1.98–20.35); for *PPP2R1A*mu/*FBXW7*mu (using *CA2*+*E2F* <4.75 as the reference), the hazard ratio was 8.90 (95% CI, 2.50–31.67; *P* = .002). These results suggest that *ECPPF* profiles patients with early-stage, low-risk EEC who have substantial risk of occult extrauterine disease and are candidates for early therapeutic intervention.

### Association of MHR *ECPPF* with compromised survival in *CTNNB1*mu EEC

*CTNNB1*mu in low-risk EEC is reportedly associated with compromised survival [32]. *CTNNB1*mu were identified in 28 patients with early-stage, low-risk EEC (stage I, G1 or G2, ≤50% MI). Seven patients (25%) had documented recurrences, and 6 of these recurrences occurred among the 9 patients in this group with MHR. *ECPPF* status of patients with early-stage, low-risk *CTNNB1*mu was associated with differences in survival (*P* < .001) (Fig 3C). The *CTNNB1*mu cohort was expanded to include all stage I and II, G1 and G2 cases. Among these 39 cases, 95% of missense mutations were in exons 4 and 5 of the *CTNNB1* gene. *ECPPF* efficiently stratified outcomes (*P* < .001); the hazard ratio (95% CI) for MHR vs MLR was 19.58 (2.38–161.04; *P* = .006). These results further underscore the ability of *ECPPF* to distinguish high-risk patients traditionally thought to have low-risk EEC.

### Association of MHR *ECPPF* with therapeutic insensitivity in patients with high-risk EEC

In the absence of HRmu, the markedly different expression of cell cycle and HR genes in EEC (Table 2) predicts disparate responses to PbCT and radiotherapy [15–17]. Among 40 patients with stage II to IV EEC, 10 therapeutic failures (25%) were documented. Two failures occurred among 21 patients (9.5%) with MLR, and 8 occurred among 19 patients (42.1%) with MHR.

Patients with more advanced EEC (stages III-IV; stages I-II, G2, >75% MI; and stages I-II, G3, >50% MI) invariably receive adjuvant PbCT or radiotherapy (or both). Significant divergence in PFS were observed in this broad at-risk cohort (*P* = .006) and for patients with stages III to IV disease alone (*P* = .002) as a function of MLR and MHR *ECPPF* (Fig 3D and 3E). These results suggest that EEC with MLR *ECPPF* is responsive to PbCT or radiotherapy (or both), whereas MHR *ECPPF* likely is insensitive.

### Association of MLR *ECPPF* and MHR *ECPPF* plus HRmu with favorable prognoses

**Fig 4:**
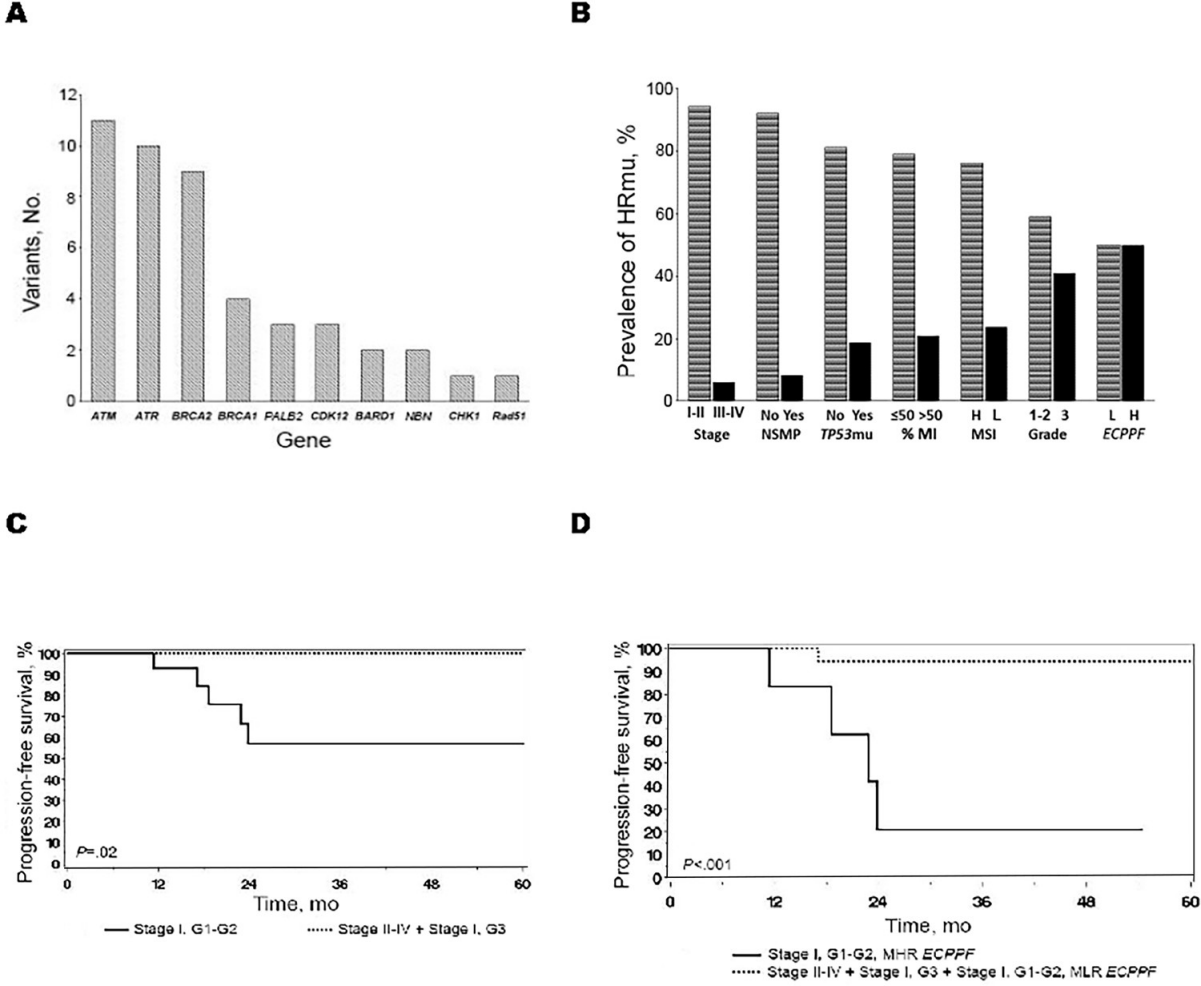
Homologous recombination repair mutations (HRmu) and *ECPPF* integration. A, Prevalence of the 34 most common HRmu (excluding *POLE*mu) detected among 164 endometrioid endometrial cancers. B, Prevalence of HRmu according to stage, no specific molecular profile (NSMP), *TP53*mu, myometrial invasion (MI), microsatellite instability (MSI) high (H) or low (L), grade (G), and *ECPPF*. C, Progression-free survival among 34 HRmu cases stratified according to traditional risk categories: low risk was defined as stage I, G1-G2 (n = 16), and high risk was defined as stage I, G3 and stages II-IV (n = 18). D, Progression-free survival among 34 patients with HRmu, stratified by stage and *ECPPF* molecular low risk (MLR) and molecular high risk (MHR).

HRmu were detected in 34 patients (20.7%), with 7 (20.6%) harboring more than 1 HRmu. *ATM*mu, *ATR*mu, and *BRCA2*mu were the most prevalent variants (Fig 4A); 94% of mutations occurred in stages I to II EEC and 76% were classified as MSI-H (Fig 4B). No therapeutic failures were documented in the 18 at-risk patients with HRmu (stage I, G3 [n = 13]; stage II [n = 3]; stage III [n = 1]; and stage IV [n = 1]) who frequently received adjuvant treatment; this group included 12 (66%) with MHR *ECPPF* (Fig 4C). In contrast, recurrences were documented for 5 of 16 patients (31%) with stage I, G1 to G2 disease, who are seldom candidates for adjuvant therapy; 4 had invasion only into the inner third of the myometrium but 4 also were MHR *ECPPF*. Among 28 at-risk patients with HRmu (likely candidates for adjuvant therapy) and MLR *ECPPF* (likely untreated cases), 1 therapeutic failure (3.6%) was documented, whereas for the 6 patients with MHR *ECPPF* (generally managed with adjuvant therapy), 4 recurrences (66%) were documented (*P* < .001) (Fig 4D). Collectively, these results suggest that MHR *ECPPF* with HRmu and MLR *ECPPF* EEC are sensitive to PbCT or radiotherapy (or both). Patients with MHR *ECPPF* and HRmu, including those with early-stage, low-grade EEC, must be identified so that they can receive definitive treatment.

## Discussion

This study provides evidence that the *ECPPF* molecular classifier can profile patients with low-risk stage I, G1 or G2 EEC, including *CTNNB1*mu, with substantial risk of recurrence who would benefit from adjuvant therapy. *ECPPF* also profiles subgroups of patients with high-risk and advanced-stage EEC who have distinct responses to PbCT or radiotherapy (or both). Lastly, *ECPPF* associates with outcomes in a manner independent of traditional risk factors (eg, *TP53*mu, *CTNNB1*mu), except for stage IV disease. *CCNA2*-L/*E2F1*-L was associated with excellent PFS rates across traditional risk categories, paralleling survival rates associated with *POLE*mu. In contrast, *CCNA2*-H/*E2F1*-H, *PPP2R1A*mu, and/or *FBXW7*mu were associated with significantly compromised recurrence-free survival among patients with low- and high-risk EEC, suggesting that patients with MHR *ECPPF* likely are insensitive to adjuvant therapies (ie, radiotherapy and/or PbCT).

On the basis of our previous study [18], we postulated that treatment failures in MHR *ECPPF*, predominantly among patients with HR-proficient EEC, would correlate with overexpression of cell cycle and HR regulatory components, which are hallmarks of resistance to radiotherapy and PbCT [16, 17]. The differential expression of multiple cell cycle and HR genes among patients with *CCNA2*-L/*E2F1*-L vs *CCNA2*-H/*E2F1*-H was substantial (*P*<1.0E-10). The large disparity in expression potentially suggests that a functional deficiency in HR may exist and is likely caused by attenuated expression of cell cycle and HR genes in *CCNA2*-L/*E2F1*-L [29–31]. This mechanism may account for their favorable responses to PbCT and radiotherapy, even among patients with G3 and advanced-stage EEC. Notably, clinicopathologic characteristics and cell cycle and HR expression profiles for *PPP2R1A*mu and *FBXW7*mu mirrored those of *CCNA2*-L/*E2F1*-L, but PFS rates were strikingly different, suggesting distinctly diverse oncogenic mechanisms. In contrast, the substantially increased expression of cell cycle and HR genes in *CCNA2*-H/*E2F1*-H implies enhanced DNA damage response mechanisms, which is consistent with their frequent lack of sensitivity to PbCT or radiotherapy (or both) [16, 17]. Considering that FOXM1 is reported to induce multiple HR genes [21, 23], the overexpression of *FOXM1* and lack of FOXM1 degradation (due to *CIP2A* overexpression) likely are contributing to greater expression of HR genes [18, 26].

In this study, *ATM*, *ATR*, *BRCA2*, and *BRCA1* were the most prevalent HRmu and predominantly were observed with stage I to II, low-grade EEC. Almost no recurrences were observed among patients with high-risk, stage I EEC, patients with advanced-stage EEC with HRmu, and patients with stage I, G1 to G2, MLR EEC who were unlikely to receive adjuvant treatment. In contrast, the treatment failure rate was high among patients who were MHR *ECPPF* and HRwt, regardless of stage or grade. Considering that HRmu EEC appeared sensitive to adjuvant therapies, identification of MLR and MHR *ECPPF* with HRmu would facilitate selection of patients who are candidates for molecular-based therapeutic approaches. The molecular distillate from this study suggests that treatment regimens with PbCT or radiotherapy (or both) are efficacious for patients with MLR and MHR *ECPPF* plus HRmu, but they lack acceptable therapeutic efficacy for patients with MHR *ECPPF* plus HRwt. Patients with MHR *ECPPF* plus HRwt, including those with stage I disease, appear to be preferable candidates for innovative phase 1 or 2 clinical trials, regardless of traditional clinicopathologic risk factors.

Mechanistically, CCNA2, E2F1, PP2A, and FBXW7 are pivotal determinants of cell cycle, DDR, and PI3K-AKT signaling dysregulation [18, 20–26]. With CCNA2 upregulation, E2F1 is reportedly converted to a potent transcription activator of FOXM1, CIP2A, cell cycle, and HR genes [19–22]. A previous study from our group provided a schematic to describe the mechanistic integration of projected CCNA2-E2F1-CIP2A axis targets and signaling pathways [18]. The consequent induction of HR genes by FOXM1, coupled with suppressed degradation of FOXM1 and cell cycle proteins (from CIP2A inhibition of PP2A), is consistent with enhanced DDR and insensitivity to DNA-damaging agents [23, 24]. *PPP2R1A*mu and *FBXW7*mu likewise markedly impede degradation of these proteins [24, 25, 33]. Navigation of these mechanisms helps characterize EEC molecular vulnerabilities and allows speculation about potential innovative therapeutic options. Such options may include modulators of the CCNA2-E2F1-CIP2A axis (eg, PRMT5 or CIP2A inhibitors), activators of *PPP2R1A*mu/PP2A, and inhibitors of downstream FBW7/E3 ubiquitin ligase substrates (eg, DDR elements) [33–36].

Limitations of this study include variation in the duration of short- and long-term surveillance, which thereby limited the survival analyses to progression-free intervals. Long-term PFS is likely underestimated, considering that the median surveillance period for recurrence-free cases was less than 30 months and the lower quartile was 17 months. Nevertheless, most patients had early-stage, low-grade EEC, and an unexpected and sizable number of them had documented recurrences, which allowed us to identify significant, disparate molecular associations. Unfortunately, sites of recurrence were not annotated. Additionally, we could not evaluate PFS in the context of specific therapeutic interventions because the details of adjuvant therapies were not available. However, the submitted specimens were predominantly from gynecologic oncology services at comprehensive cancer centers, where standards of care for EEC are well defined. The predominant adjuvant therapies during patient accrual were PbCT or radiotherapy (or both); 98% of patients receiving chemotherapy received PbCT [5]. Strengths of the study include the size of the population, central pathology review, and the robust genomic and transcriptomic analyses that facilitated integration of specific molecular aberrations with clinicopathologic risk factors and adverse events.

In summary, MLR *ECPPF* and HRmu were associated with therapeutic efficacy in EEC, and MHR *ECPPF* was associated with low-risk, early-stage recurrences and insensitivity to current adjuvant therapies. Consequently, patients with MHR *ECPPF* plus HRwt EEC, including those with stage I disease, appear to be candidates for phase 1 or 2 clinical trials evaluating innovative, molecular, target-specific primary therapies.

## Abbreviations and gene expansions

ARID1A: AT-rich interactive domain-containing protein 1A
ATM: ataxia-telangiectasia protein
ATR: ataxia telangiectasia and Rad3-related
BARD1: BRCA1-associated RING domain protein 1
BRCA1: breast cancer type 1 susceptibility protein
BRCA2: breast cancer type 2 susceptibility protein
*CA2*+*E2F*: quantitative combined expression (log_2_) of *CCNA2* and *E2F1*
CCNA2: cyclin A2
CDK12: cyclin-dependent kinase 12
CHK1: checkpoint kinase 1
CIP2A: cancerous inhibitor of protein phosphatase 2A
CNH: copy number high
CNL: copy number low
CTNNB1: catenin β1
DDR: DNA damage repair
EC: endometrial cancer
EEC: endometrioid endometrial cancer
ECPPF: *E**2F1*, *C**CNA2*, *P**OLE*, *P**PP2R1A*, *F**BXW7*
E2F1: E2F transcription factor 1
FBW7 or FBXW7: F-box and WD40 domain protein 7
FOXM1: Forkhead box M1
G: grade
-H: high
HR: homologous recombination repair
KRAS: Kirsten rat sarcoma virus
-L: low
MHR: molecular high-risk *ECPPF*
MI: myometrial invasion
MLR: molecular low-risk *ECPPF*
MSI-H: high microsatellite instability
-mu: mutation(s)
NBN: nibrin
NSMP: no specific molecular profile
PALB2: partner and localizer of BRCA2
PbCT: platinum-based chemotherapy
PFS: progression-free survival
PI3K-AKT: phosphatidylinositol 3-kinase−protein kinase B
PIK3CA: phosphatidylinositol-4,5-bisphosphate 3-kinase catalytic subunit α
PIK3R1: phosphatidylinositol 3-kinase regulatory subunit α
POLE: DNA polymerase e, catalytic subunit
PPP2R1A: protein phosphatase 2 scaffold subunit α
PP2A: protein phosphatase 2A
PRMT5: protein arginine methyltransferase 5
PTEN: phosphatase and tensin homologue
Rad51: Rad51 recombinase
SEC: serous endometrial cancer
TCGA: The Cancer Genome Atlas
TP53: tumor protein p53
-wt: wild type
